# Notes and Notices

**Published:** 1897-06

**Authors:** 


### NOTES AND NOTICES.

DR. W. L. REED, of St. Louis, Mo., has been appointed one of the Board of Managers for Falon Asylum, No. 1. Score one for Homeopathy and the good homeopathic Governor of Missouri!

DR. A. W. PHILLIP, of Derby, Conn., President of The State Homeopathic Medical Society, has been appointed Surgeon General upon the staff of Governor Cooke.

THE COLORADO HOMEOPATHIC MEDICAL SOCIETY held its eleventh annual meeting on Tuesday and Wednesday, May 25th and 26th, 1897, in the Brown Palace Hotel in Denver.

THE MISSOURI STATE MEDICAL ASSOCIATION held its fortieth annual meeting May 18th, 1897, at the Century Theatre, St. Louis, Mo.

MIDDLETOWN HOMEOPATHIC HOSPITAL, Albany, N. Y., May 6th.—Dr. Charles Spencer Kinney has been appointed, under civil service rules, to be First Assistant Physician at the Middletown (N. Y.) State Homeopathic Hospital.

THE INTERNATIONAL HAHNEMANNIAN ASSOCIATION will hold its eighteenth annual meeting at the International Hotel, Niagara Falls, New York, Tuesday, June 29th, and continue through the week.

THE HOMEOPATHIC MEDICAL SOCIETY OF THE STATE OF PENNSYLVANIA.—The on State Society of homeopathic doctors holds this year's session in the city of Scranton, September 21st, 22d, and 23d. Every "Similar" soul in the State is invited to make a pilgrimage to the 1897 shrine of our faith. You are to bring with you some illustrations of the simple means you have employed during the past year to alleviate suffering and preserve life; for which you will receive in exchange, measure for measure, that which will broaden the scope of your usefulness and better equip you for the unexpected of to-morrow. You are not to expect that everything you hear or see will be new to *you*; yet it may be new to some one less experienced. If you gather *one* truth that will enable you to cope successfully with a condition that may materialize any moment, you will return to your labors a better doctor—and the best is none too good. Dr. Gramm, the progressive Secretary, has a scheme on foot by which the business of the meeting is to be conducted in a manner conducive to the greatest expedition and evolvement of the best interests of everybody who attends. As no little space will be given to the social feature of this meeting, it is hoped that members will bring their families with them, thus adding the strength that ever prevails where numbers congregate. Z. T. MILLER, M. D., President, 2013 Carson Street, Pittsburg, Pa.; EDWARD M. GRAMM, M. D., Corresponding Secretary, 1433 Girard Avenue, Philadelphia, Pa.

